# CD39 activity correlates with stage and inhibits platelet reactivity in chronic lymphocytic leukemia

**DOI:** 10.1186/1479-5876-5-23

**Published:** 2007-05-04

**Authors:** Dianne Pulte, Kim E Olson, M Johan Broekman, Naziba Islam, Harold S Ballard, Richard R Furman, Ashley E Olson, Aaron J Marcus

**Affiliations:** 1 Research Service, Veterans Affairs New York Harbor Healthcare System, New York, NY 10010, USA; 2 Medicine-Hematology/Oncology, Weill Medical College Cornell University, New York, NY 10021, USA; 3 Medical Service, VA NY Harbor Healtcare System, New York, NY 10010, USA; 4 Pathology and Laboratory Medicine, Weill Medical College of Cornell University, New York, NY 10021, USA

## Abstract

**Background:**

Chronic lymphocytic leukemia (CLL) is characterized by accumulation of mature appearing lymphocytes and is rarely complicated by thrombosis. One possible explanation for the paucity of thrombotic events in these patients may be the presence of the ecto-nucleotidase CD39/NTDPase-1 on the surface of the malignant cells in CLL. CD39 is the major promoter of platelet inhibition *in vivo *via its metabolism of ADP to AMP. We hypothesize that if CD39 is observed on CLL cells, then patients with CLL may be relatively protected against platelet aggregation and recruitment and that CD39 may have other effects on CLL, including modulation of the disease, via its metabolism of ATP.

**Methods:**

Normal and malignant lymphocytes were isolated from whole blood from patients with CLL and healthy volunteers. Enzyme activity was measured via radio-TLC assay and expression via FACS. Semi-quantititative RT-PCR for CD39 splice variants and platelet function tests were performed on several samples.

**Results:**

Functional assays demonstrated that ADPase and ATPase activities were much higher in CLL cells than in total lymphocytes from the normal population on a per cell basis (p-value < 0.00001). CD39 activity was elevated in stage 0–2 CLL compared to stage 3–4 (p < 0.01). FACS of lymphocytes demonstrated CD39 expression on > 90% of normal and malignant B-lymphocytes and ~8% of normal T-lymphocytes. RT-PCR showed increased full length CD39 and splice variant 1.5, but decreased variant 1.3 in CLL cells. Platelet function tests showed inhibition of platelet activation and recruitment to ADP by CLL cells.

**Conclusion:**

CD39 is expressed and active on CLL cells. Enzyme activity is higher in earlier stages of CLL and decreased enzyme activity may be associated with worsening disease. These results suggest that CD39 may play a role in the pathogenesis of malignancy and protect CLL patients from thrombotic events.

## Background

Chronic lymphocytic leukemia (CLL) is one of the most common leukemias in North America, representing 25–30% of all leukemias, with 10,020 new cases and 4660 deaths estimated for 2006 [1,2]. The leukemia is characterized by a clonal expansion of long-lived, mature appearing B-lymphocytes that co-express the CD5, CD19, and CD23 surface antigens [3]. In contrast to many other malignancies, CLL is not known to be associated with an increased risk of thrombosis [4]. Patients with thrombosis in CLL have been reported, but most occur in association with acquired inhibitors of anti-thrombotic moieties [4-6]. Conversely, there are only a few case reports in the literature of abnormal hemorrhage in CLL patients [7-9]. In addition, successful completion of pregnancies uncomplicated by thrombotic events have been reported in patients with CLL [10,11], suggesting that these patients can withstand pro-thrombotic states. Two recent studies of cell surface molecules in CLL show that they express many of the same markers as activated normal B-cells including CD39 [12,13]. Although CLL cells resemble activated B-cells, they function more like anergized B-lymphocytes. Therefore, some of the cell surface markers observed usually associated with activated B-cells may be inactive in CLL. No studies of the activity of CD39 in CLL have been performed to date.

CD39 (ecto-nucleotidase, NTDPase1), is an ADPase and ATPase found on the surface of endothelial cells, normal lymphocytes, and other leukocytes [14,15]. It is strongly expressed on peripheral B-lymphocytes, weakly expressed on marginal zone B-lymphocytes, but not on B-lymphocytes in germinal centers [16,17]. Its principal function on the endothelial cell surface is to decrease platelet activation and recruitment by metabolizing platelet-released ADP. In leukocytes, the enzyme has a variety of other effects as well, including modulation of cytokine expression and the inflammatory response [18,19], as well as cell-cell adhesion [14]. CD39 will quickly metabolize the ADP produced by its metabolism of ATP to AMP. The enzyme is usually more efficient in metabolizing ADP than ATP [20]. Five apyrase conserved regions on the molecule are essential for preservation of CD39 enzyme activity [21].

A decreased ratio of ADPase: ATPase activity in lymphocyte CD39 has been reported in patients with coronary artery disease [22], suggesting that inefficient or aberrant CD39 activity may be involved in the pathogenesis of arterial vascular disease. Variation of the ADPase:ATPase ratio has been observed between individuals, although intra-personal variation has not yet been observed. Although very low levels of CD39 expression on platelets has been reported [23,24], our laboratory found no difference between ATPase and ADPase activity in platelet-rich and platelet-poor plasma. This suggests that the contribution of platelet CD39 is minimal to non-existent and that leukocyte and endothelial cell CD39 exert primary control of ATP and ADP conversion to ADP and AMP (unpublished data). Pharmacological data in animals suggests that supra-physiologic levels of CD39 are not associated with clinical hemorrhagic episodes [25] Transgenic mice which over-expressed CD39 showed a prolonged bleeding time but did not show overt bleeding tendencies and had no increase in bleeding associated with parturition or routine experimental procedures. Additionally, these mice were protected against thrombosis induced by injection of ADP or collagen [23]. CD39 has also been shown in a murine model of xengraft rejection to be protective against early rejection by reducing platelet reactivity [23].

Aberrations in CD39 expression have been observed in other neoplastic disorders. One publication noted increased expression of CD39 *in vitro *in a differentiated human melanoma cell line as compared with normal melanocytes. Decreased CD39 expression was seen in less differentiated sub-clones, with the least differentiated cell lines expressing far less activity than normal melanocytes [26]. A study of CD39 activity in pancreatic cancer reported decreased activity in smooth muscle cells surrounding the ducts in the malignant tissue as compared with normal pancreatic tissue [27]. An *in vitro *study of myeloid cancer lines found that CD39 was not expressed on myeloblasts, but was expressed on later myeloid cell lines [28]. This finding is particularly interesting as acute myeloid leukemia is associated with increased thrombosis, whereas chronic myeloid leukemia is less so[29,30]. In this report, we studied the role of CD39 in CLL and considered the implications of this role in both CLL and vascular biology in general.

## Methods

### Patient population

Patients were recruited from the hematology clinic and inpatient hematology/oncology service at the VA Harbor Healthcare System and Weill Medical College of Cornell University. The protocol was approved by the Institutional Review Boards at both institutions. The diagnosis of CLL was made by the presence of the characteristic immunophenotype (CD5+, CD19+, CD23+, sIg dim). Patients with CLL were asked to participate in the study if they were able to give informed consent, had a hemoglobin of > 8 and had no contraindications to a blood donation. After informed consent was obtained, 10–50 ml of whole blood was withdrawn via free flow from each donor. Blood was drawn from healthy volunteers to be used as a control population.

### Lymphocyte isolation

Lymphocytes were isolated according to a modified histopaque isolation protocol. Whole blood was centrifuged and platelet-rich plasma removed. Phosphate buffered saline (PBS) was added to the remaining blood to restore the original plasma volume. Blood was then diluted 1:1 with PBS and carefully layered on a histopaque gradient. Samples were centrifuged for 30 minutes at 820 g, the buffy coat isolated and washed with PBS × 3. The cells were then counted. In cases in which > 90% lymphocytes were present, no further isolation was needed. In cases in which < 90% lymphocytes were present, cells were incubated in RPMI with 10% fetal calf serum (FCS) for 30 minutes × 2 to deplete monocytes, which adhered to the bottom of the flask. Lymphocytes remaining in fluid phase were washed with PBS, and re-counted. Short term FCS exposure (< 24 hours) did not affect CD39 expression or activity. Cell purity as assessed by FACS was > 80% CLL lymphocytes. In cases where the purity of CLL cells was less than 80%, an alternate isolation technique, described below was used.

B-lymphocytes were isolated using the RosetteSep (StemCell, Vancouver) B-lymphocyte isolation kit per the manufacturer's protocol. The RosetteSep B-lymphocyte isolation kit uses bispecific antibodies against CD2, CD3, CD16, CD36, CD56, CD66b, and glycophorin A to deplete non-B-lymphocytes from the histopaque layer. Since the antibody cocktail does not include CD5, it should not deplete CLL lymphocytes. B-lymphocytes were isolated from controls and from two patients who had small CLL clones. In all other cases, > 80% of total lymphocytes were malignant and therefore CD39 activity in total lymphocytes was considered to be the equivalent of activity in malignant lymphocytes.

### Assay for ADPase and ATPase activity/TLC

To assay CD39 activity, cell suspensions were incubated with 50 μmolar ^14^C-labeled ADP or ATP in 50 μliter assay buffer for five minutes and the reaction terminated by addition of a "stop solution". The stop solution consisted of 160 mM EDTA at a pH of 7 and 17 mM ADP in 0.9% saline[31]. Suspended cells were then removed by centrifugation and the supernatant decanted for analysis of the reaction. Thin-layer chromatography (TLC) was used to separate labeled nucleotides, nucleosides, and bases. Their radioactivity was then analyzed to assess metabolism of ^14^C-labeled ADP or ATP by CD39. Data are expressed as a percentage of ADP or ATP metabolized or as picomoles nucleotide metabolized per minute per 50,000 or 100,000 cells. This TLC system was developed in our laboratory [22,31,32].

### FACS analysis

Cells were single stained with CD3-APC, CD19-FITC, CD39-PE, CD4-PERCP, and CD8-FITC (CD19-FITC and CD39-PE Ancell, all others BD Biosciences). The CD39 antibody used was BU-61, an antibody that identifies full-length CD39 and some, but not all of the splice variants identified (see below). The splice variants we identified do not occur in isolation, but are always associated with full-length CD39. Therefore, it is unlikely that the percentage positive cells would change if another antibody were used, although there could be some change in the per cell density identified by FACS. Cells were also double stained for CD3/CD39, CD4/CD39, CD8/CD39, and CD19/CD39. Cells plus antibody were incubated for 45–90 minutes at room temperature with shaking. Acquisition and analysis of FACS data was performed on FACSCanto (BD Biosciences) using FACSDiva software (BD Biosciences). Percentage positivity and geometric mean fluorescence for normal and malignant lymphocytes were examined.

### Isolation of RNA and conversion to cDNA

Cells remaining after activity assay were pelleted and frozen at -70°C. RNA was isolated via Trizol (Invitrogen) per manufacturer's protocol and cDNA produced by incubation with reverse transcriptase.

### Semiquantitative RT-PCR

cDNA from patients and normal controls was produced as above and evaluated for CD39 by semi-quantitative PCR. We used primers that identify splice variants as well as full length CD39 to determine whether any differences in expression of splice variants were present between normal lymphocytes and CLL cells.

### Platelet reactivity studies

Platelet-rich plasma (PRP) and lymphocytes were prepared from patients with CLL. PRP was prepared by centrifuging whole blood from patients at 200 g for 15 minutes, removing the plasma layer, and re-centrifuging this at 90 g for 10 minutes to remove any residual erythrocytes. Platelet-poor plasma was prepared by centrifuging an aliquot of PRP at 3000 g for 3 minutes in an Eppendorf centrifuge and removing the supernatant. Lymphocytes were prepared as above. Platelet aggregation curves were developed with PRP, using ADP at 1, 2, 5, and 10 μM. 0.5–1 × 10^6 ^CLL lymphocytes were then added to PRP and platelet-poor plasma (PPP) controls. The aggregation response to ADP was measured and recorded [21,33].

### Statistics

Student's two sided T-test with unequal variance was used to generate p-values when comparing groups.

## Results

### Patient clinical data

Blood samples from 21 patients (3 women, 18 men) from the two institutions were drawn for this study. Three patients underwent more than one blood donation.

Few of the patients had a history of thrombotic events or hemorrhage before or after the diagnosis of CLL was made. One patient had a history of stroke diagnosed prior to the onset of CLL and one had a history of heart disease and deep venous thrombosis prior to his diagnosis. Neither patient had any further events after diagnosis. Patient characteristics are summarized in Table 1.

**Table 1:** Clinical characteristics of patients studied

Age		71.5 y/o (35–95 y/o)
Gender	Male	18
	Female	3
Date of diagnosis		1990–2005
Stage	0	9
	1	2
	2	2
	3–4	8
History of chemotherapy	Yes	10
	No	11

Clinical characteristics of patients in this study. A gender imbalance is seen because the majority of patients were recruited from the VA hospital system.

Total lymphocytes were isolated from 22 healthy volunteers (9 women, 13 men) for use as a control population. In preliminary studies, it became clear that CD39 expression varied between B-lymphocytes and T-lymphocytes. Therefore, B-lymphocytes were isolated from an additional 8 volunteers. None of the normal donors had a history of thrombotic events or hemorrhage.

### Radio-TLC data

ADPase and ATPase activities were observed in malignant lymphocytes from all patients. However, the level of activity varied greatly. ADPase ranged from 4.3 to 1263 pmol/min/50 K cells and ATPase ranged from 7.2 to 70 pmol/min/50 K cells. Greater ADPase activity was seen in patients with stage 0–2 disease (p = 0.002), hemoglobin greater than 13 (p = 0.03), and patients who were over 70 years of age (p = 0.002). A trend toward lower activity was seen in patients who had undergone chemotherapy and those with leukocyte counts of greater than 20 × 10^3 ^per μl, but the p-values were non-significant (p = 0.08 and 0.15 respectively), possibly due to the small sample size (Table 2). Because of the relatively small numbers of patients studied thus far, we were unable to perform multivariate analysis and so cannot make definitive statements as to whether all the variables identified are independent. No correlation between gender, ethnic origin, platelet count, or duration of disease and CD39 activity was observed. In controls, CD39 activity did not vary with age, gender, ethnic origin, or any hematologic parameter. In a previous study, decreased CD39 activity was observed in patients with heart disease and hypertension but no correlation with age was observed (18).

**Table 2:** Characteristics associated with changes in CD39 function

	Characteristics	Activity (pmol/min/50 K cells)
Stage	0–2	88.2*
	3–4	44.7
History of chemotherapy?	Yes	55.8
	No	83**
Age	< = 70 y/o	46.5*
	> 70 y/o	92
Hemoglobin	< 13 g/dl	57.9^†^
	> = 13 g/dl	89.4

Statistically significant differences in CD39 activity. Stage, age, and hemoglobin levels were associated with statistically significant differences in the enzymatic activity of CD39 in patients with CLL. A history of chemotherapy was associated with a marginally significant trend towards decreased CD39 activity in CLL. Leukocyte and platelet count, gender, and length of time since diagnosis were not associated with differences in CD39 activity.

*p = 0.002

**p = 0.08

^†^p = 0.03

Repeat sampling was performed when clinical indications of worsening disease were observed. Samples were collected prior to institution of therapy. Among the patients who had repeat sampling after worsening of disease, two of three had decreased CD39 activity. The other patient had decreased ATPase activity, but slightly increased ADPase activity. Interestingly, this patient had an increase in the density of CD39 expression per cell as observed by FACS of about double (see below), suggesting that activity per molecule decreased in this patient as well.

**Figure 1:**
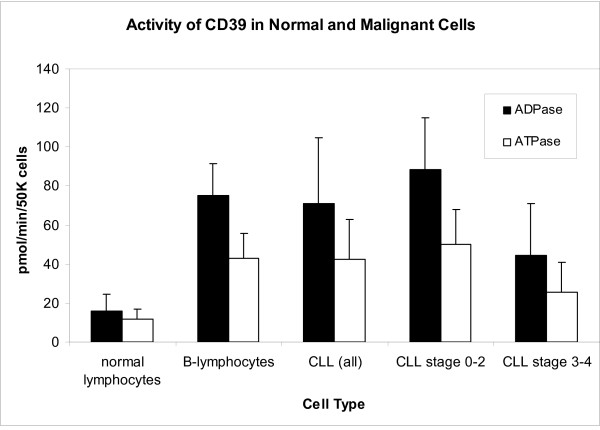
CD39 activity as measured in normal and malignant lymphocytes. The enzyme was measured by radio-TLC as described in methods. ADPase and ATPase activity was elevated in cells of all CLL patients, early (stage 0–2) CLL, and late (stage 3–4) CLL as compared to normal lymphocytes (p < 0.00001 for all and early CLL versus normal lymphocytes; p < 0.05 for late CLL). ADPase activity was elevated in early CLL versus normal B-lymphocytes (p = 0.05) but decreased in late CLL (p < 0.01).

CD39 activity was much greater in CLL cells than in normal lymphocytes. However, CLL is a disease of B-lymphocytes and we have found that normal B-lymphocytes express CD39 at higher levels and on a larger percentage of cells than T-lymphocytes. Therefore, B-lymphocytes were isolated from 8 normal controls and compared to the malignant lymphocytes from CLL patients. No significant difference was found between CD39 activity in normal B-lymphocytes and malignant lymphocytes in CLL patients. However, normal B-lymphocytes showed mildly decreased activity compared to stage 0–2 CLL and increased activity compared to stage 3–4 CLL (Figure 1). Lack of the IgVH gene mutation is a known poor prognostic factor in CLL. It is possible that both lack of the IgVH mutation and low CD39 activity result from malignant transformation of less mature cells. However, this does not explain all of the differences in CD39 activity between early and later stage CLL since IgVH mutational status does not change throughout the course of the disease.

### FACS data

FACS for CD39 was obtained on 14 patients and 8 controls. As expected, patients showed a much higher percentage of CD39 expression on total lymphocytes (71 versus 21%, p < 10^-7^). Expression on normal B-lymphocytes versus all CLL cells did not differ significantly (93% versus 95%, p = 0.5). CD39 expression was greater in CLL patients who had not received chemotherapy than in those who had (99% versus 89%), but the difference was not statistically significant (p = 0.1), perhaps because of a wider variation in CD39 expression in CLL cells as opposed to normal B-lymphocytes.

Mean per cell expression of CD39 was similar on CLL cells as compared to normal B-lymphocytes (Table 3). However, the range of expression on CLL cells was extremely large, with a geometric mean ranging from 600 to 10,750 in CLL, versus 2100–4600 in normal B-lymphocytes, possibly indicating the presence of multiple sub-groups, some of which had higher levels of expression, others lower. Interestingly, expression of CD39 by FACS was not always well correlated with CD39 function as measured by radio-TLC. Two patients who progressed during the study had FACS measurement of CD39 levels both before and after progression of the disease. In both cases, the percentage cells positive for CD39 was essentially unchanged. However, in the first instance, the geometric mean decreased by approximately a factor of four, whereas in the second, the geometric mean actually increased by 2–3 times. ADPase and ATPase function decreased by about 50% in the first case, but ADPase was slightly increased in the second although ATPase decreased slightly in the setting of increased CD39 expression. The reason for this difference is not clear from current information, although the observation suggests that CD39 may become less functional as CLL evolves, even in cases where its expression is unaltered or even increased.

**Table 3:** CD39 expression on sub-types of normal lymphocytes

Lymphocyte sub-type	Average % CD39+ cells (range)	Mean expression of CD39 per cell
Total normal lymphocytes	20* (14–23)	2463** (1800–3000)
T-lymphocytes (CD3+)	8.2* (1.2–15)	1717* (900–2700)
CD4+	9.1* (1.8–16)	1942* (1300–2700)
CD8+	7.9* (0.7–22)	949*^,† ^(400–1500)
B-lymphocytes (CD19+)	95 (87–99)	3640 (2100–5000)
Malignant B-lymphocytes	93 (61–100)	3480 (600–9000)

CD39 expression on normal lymphocytes. Results from FACS analysis of blood from 10 normal volunteers. Percentage CD39 expression is expressed as double positive cells as a percentage of the subtype of cells.

*p < 0.00001 for 2 tailed T-test versus normal B-lymphocytes

**p < 0.001 for 2 tailed T-test versus normal B-lymphocytes

^†^p < 0.0001 for 2-tailed T-test versus CD4+ lymphocytes

**Figure 2:**
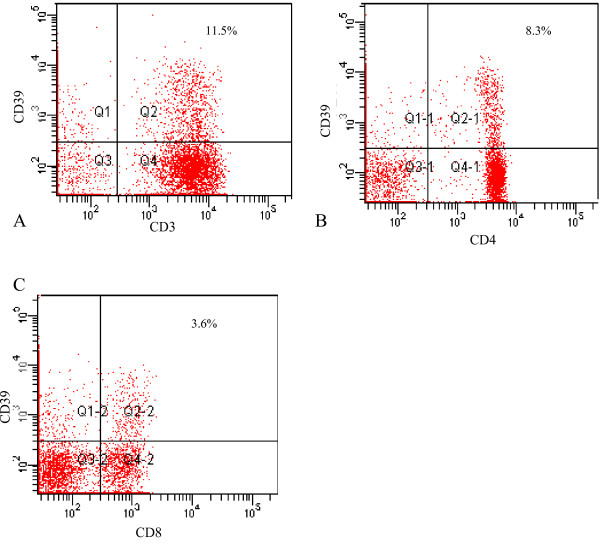
FACS of CD39 in normal T-lymphocytes. Illustrations demonstrate typical results of FACS for a lymphocyte gate. A. Total T-lymphocytes. Lymphocytes in the right upper quadrant (quadrant 2) are positive for both CD39 and CD3. B. CD4+ lymphocytes. C. CD8 positive lymphocytes.

**Figure 3:**
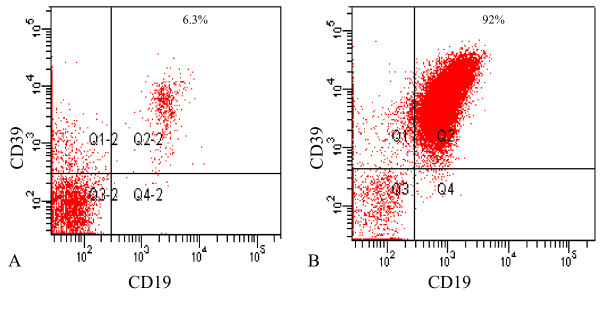
Typical FACS of normal and malignant B-lymphocytes. A. Normal B-lymphocytes B. Malignant B-lymphocytes. The majority (> 90%) of both normal and malignant B-lymphocytes are positive for CD39 and the mean expression per cell is similar. However, the number of B-lymphocytes as a percentage of the total lymphocyte gate is higher. Expression of CD39, as measured by FACS, was not clearly greater in earlier stage CLL as opposed to normal B-lymphocytes or later stage CLL, although activity was greater in earlier stage disease.

Characterization of CD39 expression on normal lymphocytes showed a variation between expression on T-lymphocytes and B-lymphocytes. Whereas over 90% of B-lymphocytes express CD39, only a minority (~8% of CD3+ cells) of T-lymphocytes expressed the enzyme. Expression in CD4+ cells and CD8+ cells was similar (Table 3). Geometric mean expression in normal T-lymphocytes tended to be lower than expression in normal B-lymphocytes and in CD8+ cells compared to CD4+ cells (Table 3). FACS data in normal and malignant lymphocytes is summarized in Figures 2 and 3.

### RT-PCR

We have recently identified two splice variants of CD39 in addition to the full length form of the molecule. One of these splice variants, called 1.5, has been shown to decrease the activity of CD39 when co-expressed with full length CD39 in COS cells [34]. The effects of the other variant (1.3), are less clear, but it may enhance CD39 activity when co-expressed with full length CD39 with or without variant 1.5[34]. Normal T-lymphocytes express high levels of full length CD39 and variable amounts of variants 1.3 and 1.5 on a per positive cell basis. B-lymphocytes express relatively high levels of 1.3 compared to T-lymphocytes or malignant B-lymphocytes. Semi-quantitative RT-PCR on CLL lymphocytes showed increased expression of 1.5 and full length CD39 and decreased expression of 1.3 as compared to normal B-lymphocytes. This unusual ratio of 1.3 to 1.5 may explain the relatively low levels of enzyme activity as compared to the higher levels of antigen observed in some CLL lymphocytes. Interestingly, the antibody used for FACS, BU61, identifies full length CD39 and 1.5, but not 1.3, thus, CD39 density on CLL cells may be slightly overestimated by FACS relative to normal lymphocytes. However, splice variants occur only in complexes with full length CD39. Therefore, whereas per cell density may be slightly overestimated by FACS, percentage positivity would be unaffected.

### Platelet reactivity in CLL

**Figure 4:**
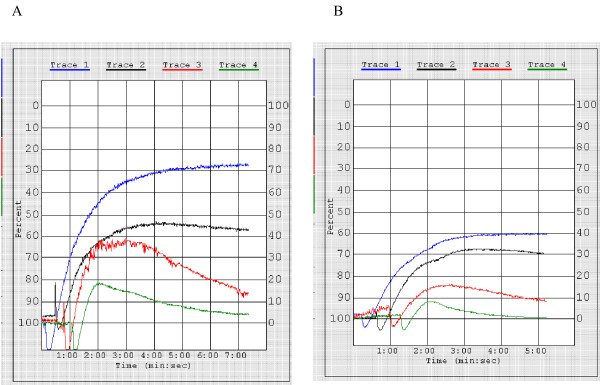
Typical results of platelet activation and recruitment as studied *in vitro *by platelet aggregometry. Figures represent aggregation curves following addition of 10, 5, 2, and 1 μM ADP to A. PRP B PRP+10^6 ^CLL cells. An equal number of CLL cells was added to platelet poor plasma (PPP) in the latter test. Area under the curve was 250 units for 10 μM ADP, 165 for 5 μM, 152 for 2 μM, and 51 for 1 μM ADP in the control curve and 170, 119, 26, and 7 units respectively for samples in which CLL cells had been added, indicating a significant decrease in the degree of aggregation when these lymphocytes were added.

**Figure 5:**
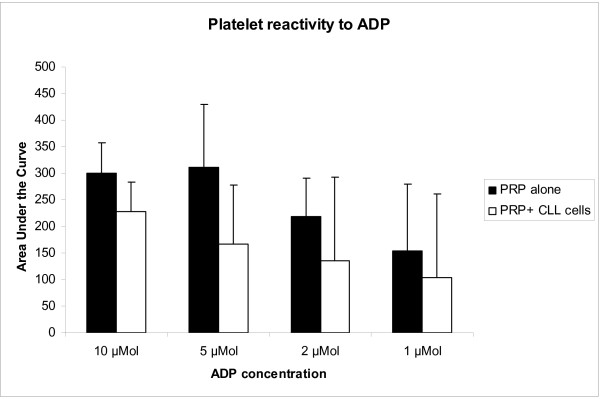
Platelet reactivity to ADP with and without addition of CLL cells. Platelet function tests were performed on platelets from six patients with CLL. Evidence of platelet hyper-reactivity with increased area under the curve (AUC) and little decrement in platelet response to ADP from 10 μM to 2 μM was seen in three out of six patients. Some decrease in platelet reactivity was seen in all patients when 5 × 10^5^-10^6 ^CLL cells were added to PRP and PPP. However, the decrease was minimal in one patient. P = 0.02 for 5 μM concentration, p = 0.1 for all other concentrations. However, data is partial for all concentrations except 5 μM. Of the six patients tested, four were stage 0, one stage 2, and one stage 3 disease. All had platelet counts of greater than 150 K.

Platelets from patients with CLL showed normal or exaggerated aggregation in response to ADP. Addition of 0.5–1 million CLL cells to PRP decreased the platelet aggregation responses to ADP in all patients tested, although the decrease was minimal in one patient (Figures 4 and 5). A preliminary dose-response study showed that addition of less than 0.5 million cells resulted in only a minimal decrease in platelet response to ADP. Limitations in the amount of cells and PRP available in this initial study precluded testing of larger quantities of cells.

Average area under the aggregation curve (AUC) for patients was > 300 for 10 and 5 μM ADP (figure 5). In contrast, average AUC in controls was 255 and 242 for 10 and 5 μM ADP (data not shown). However, two patients had AUC of > 400 at 5 μM and three had no decrement of response at 2 μM ADP, suggesting hypersensitivity in these patients. Interestingly, the patient who showed the lowest decrease in platelet response to ADP had high baseline responsiveness to ADP.

We previously demonstrated that in combined suspensions of endothelial cells and platelets, no agonist could activate or induce recruitment of platelets due to the presence of CD39. COS cell controls, which are CD39 negative, had no effect on platelet reactivity, suggesting that this is a specific property of CD39 [35].

## Discussion

We found that the majority of lymphocytes from CLL patients express active CD39 on their surface. All CLL patients had some level of CD39 activity. Overall, CLL cells had a much higher level of CD39 activity than normal lymphocytes, although their level of expression was equal to or slightly lower than that of normal B-lymphocytes when all cases of CLL were considered together. However, when CLL patients were divided into two subgroups, stages 0–2 and stages 3–4, it was found that the earlier stage CLL patients had higher CD39 activity than normal B-lymphocytes, whereas later stage CLL patients had lower CD39 activity than normal B-lymphocytes. Some patients with stage 3 and 4 disease had very low levels of activity, comparable to levels seen in normal T-lymphocytes and much lower than would be observed in normal B-lymphocytes. Thus, CD39 activity varies widely in CLL, from supra-normal levels, particularly in patients with early disease, to sub-normal levels in patients with more advanced disease. Interestingly, a similar pattern has been reported in melanoma cell lines, in which CD39 is elevated as compared to normal melanocytes in well differentiated melanoma and decreased in more advanced stages of the disease [26].

Lower levels of CD39 were associated with younger age, progression of disease, and later stage of the disorder. A trend toward lower CD39 was seen in patients who had received chemotherapy. Decreased levels of ADPase activity were observed in 2 of the 3 patients whose disease progressed during the study. In addition, decreased levels of ATPase were seen in all three. It is therefore possible that the ATPase activity of CD39 may be important in CLL over and above its anti-thrombotic effect.

A limitation of our methodologic approach is the presence of normal T- or B-lymphocytes in preparations from CLL patients. Since B-lymphocytes typically comprise 15–20% of normal lymphocytes and normal lymphocytes are a minority of the total lymphocytes in CLL patients, contamination with normal B-lymphocytes should have little effect on the overall results. Contamination with T-lymphocytes may have a greater impact in cases in which the total leukocyte count is relatively low. We attempted to minimize this potential confounding factor by carrying out B-lymphocyte isolation in patients with relatively lower leukocyte counts. Since T-lymphocytes have lower CD39 expression and activity than B-lymphocytes, the presence of varying quantities of T-lymphocytes may have led to a falsely low level of apparent CD39 activity in patients with lower leukocyte counts. A more rigorous elimination of normal lymphocytes might enhance the differences between earlier and later stages of disease and, particularly, between patients with leukocyte counts of greater or less than 20 × 10^3 ^leukocytes/μl, but it is unlikely that the results would change substantially.

Thus, CD39 may be a marker of less aggressive disease and a drop in CD39 may be indicative of progression of the disease. Further work is required to elucidate the mechanism by which CD39 acts on CLL cells. One possible mechanism is via decreased sensitivity to apoptosis in cells with lower CD39 levels. A recent publication demonstrated that dendritic cells in CD39 null mice were resistant to apoptosis due to exposure to ATP[18]. Interestingly, another study suggests that CD39 reduces apoptosis in endothelial cells, possibly by lowering ATP-induced apoptosis [36]. Preliminary data from our laboratory suggests that CD39 transfection can increase apoptosis in response to DNA damaging chemotherapy in Jurkat cells (unpublished data). We therefore hypothesize that CD39 has effects on leukocytes which differ from those on endothelial cells. Alternatively, the different splice variants may have different effects on apoptosis. Further studies in this area should clarify these questions.

Because of the much higher load of CLL cells as compared to normal B-lymphocytes, patients with CLL have a much higher total body load of CD39. This excess of CD39 may provide protection against thrombotic events by blocking platelet activation and recruitment. Thus, platelet function tests demonstrated that *in vitro*, even a relatively small number of CLL cells inhibited the platelet aggregation response to ADP. Additionally, the finding of baseline platelet hypersensitivity to ADP in two of the patients tested suggests that the patients' platelets were exposed to ADP *in vivo *to a lesser extent than normal. Therefore, the CD39 on their lymphocytes may be metabolizing ADP in their vascular environment. These findings may explain the relatively low incidence of thrombosis in patients with CLL as compared to patients with other malignancies. We plan to perform cross-over experiments in which the effects of patients' cells on normal PRP and of normal lymphocytes on patients' and normal PRP are examined to further explore this issue.

The correlation of higher CD39 levels with increasing age in CLL is unexpected, as CD39 levels in normal lymphocytes have not been observed to vary with age in this or previous studies [22]. This result may be indicative of a change in the biology of the disease in more senior patients. Others have observed that older patients with CLL are less likely to die of the disease as compared with younger patients [37]. This correlates with our observation that patients with earlier or less aggressive disease tend to have higher CD39 levels than patients with later, more aggressive disease. RT-PCR for splice variants showed that the CD39 on CLL cells has a different profile from that of CD39 on normal T- or B-lymphocytes. This profile suggests a relative down-regulation of ADPase activity in CD39 in CLL. This finding further supports the hypothesis that CD39 is involved in the pathogenesis of CLL and may explain why the abundance of CD39 in CLL patients does not produce a more dramatic reduction of the platelet response in this patient population.

## Conclusion

CD39 is expressed and active on malignant cells in CLL. The level of CD39 activity correlates with the stage of disease. A decrease in CD39 activity, though not necessarily CD39 expression, is correlated with worsening of disease. These findings suggest that CD39 may be involved in the pathogenesis of CLL and that lymphocytes in CLL may actually function as antithrombotic agents due to their CD39 content and activity.

## Competing interests

The author(s) declare that they have no competing interests.

## Authors' contributions

DP. RRF, and HSB recruited patients for the study.

DP and NI carried out the lymphocyte isolation and radio-TLC.

DP carried out FACS analysis.

KEO and AEO carried out the RT-PCR assays.

JB carried out the platelet function studies.

DP and AJM conceived of the study, participated in its design and coordination, and helped draft the manuscript.

All authors read and approved the final manuscript.
